# Novel Eco-Synthesis of PD Silver Nanoparticles: Characterization, Assessment of Its Antimicrobial and Cytotoxicity Properties

**DOI:** 10.3390/ma12233890

**Published:** 2019-11-25

**Authors:** Manal A. Awad, Manal M. Alkhulaifi, Noura S. Aldosari, Shaykha Alzahly, Ali Aldalbahi

**Affiliations:** 1 King Abdullah Institute for Nanotechnology, King Saud University, Riyadh 11451, Saudi Arabia; shaykha.alzahly@hotmail.com; 2 Department of Botany and Microbiology, College of Science, King Saud University, Riyadh 11451, Saudi Arabia; manalk@ksu.edu.sa (M.M.A.); nsaldosari@ksu.edu.sa (N.S.A.); 3 Department of Chemistry, College of Science, King Saud University, Riyadh 11451, Saudi Arabia

**Keywords:** AgNPs, pigeon droppings, TEM, EDX, antimicrobial, anticancer

## Abstract

Nanomedicine is growing due to the development of new medical diagnostic tools and new nanostructure-based therapies that exert direct biological action or function as pharmacological carriers. Nanoparticles (NPs) synthesis provides an eco-friendly approach for different applications. Among NPs, silver NPs (AgNPs) are gaining considerable research interest due to their broad range of activity and their usability in the medical and biotechnology fields. In this study, a new AgNP synthesis method was developed using an aqueous pigeon dropping (PD) extract in silver nitrate (AgNO_3_). The rapid of AgNPs yield was detected visually. Analysis of UV-vis spectroscopy, energy-dispersive X-ray spectroscopy (EDX), dynamic light scattering (DLS) and electron microscopy (TEM) transmission showed a spherical or near spherical shape of AgNPs with mean size of 135 nm. AgNPs antimicrobial activities (anti-bacterial and anti-fungal) were determined using agar well diffusion method. These NPs further screened for anticancer activity in vitro using A-549 and MCF-7 cell lines. The results showed that the inhibition zone for the obtained PD AgNPs versus *Escherichia coli*, *Pseudomonas aeruginosa*, *Staphylococcus aureus*, and *Bacillus* were 26, 18, 17 and 15 mm, respectively. PD AgNPs showed the highest antifungal effect against *Aspergillus* *flavus* and the lowest effect against *Penicillium griseofulvum*. In vitro anti-cancer activities showed that the inhibitory concentration of 50% (IC_50_) of AgNPs was 10.3 ± 1.15 and 12.19 ± 0.75 µg mL^−1^ against A-549 and MCF-7 cancer cell lines, respectively.

## 1. Introduction

Nanomaterials are exciting fields of nanoscience and nanotechnology research. Metal NPs are promising in the fields of biomedicine, engineering, chemistry and electronics [1,2]. NPs can be produced at a low cost, with high efficiency, in small sizes, on a large surface area. Among metal NPs, silver NPs (AgNPs) are characterized by their unique properties. AgNPs have been used as antiseptics, antibacterials and anti-cancer agents [3,4,5,6,7]. AgNPs have emerged as an interesting alternative approach for antimicrobial and cancer treatments, however, their toxicological effects and low biocompatibility limited their clinical applications [8,9,10]. The biosynthesis of AgNPs in the presence of plant extracts and silver ions yield high biocompatible NPs that may be handled in biomedical settings without significant adverse effects [11].

Ancient Egyptian, Greek, and Roman physicians agreed that animal excrements have valuable medical uses. For instance, camel dung and camel urine were used in Greek medicine [12,13]. Bird guano has a spongy microstructure and a undulating layered presence. This consists of an undifferentiated b-fabric light, yellowish cryptocrystalline apatite. This usually contains various types of inclusions, such as small bone fragments dissolved, insect scales and plant remains, depending on the bird’s diet. The diet of pigeons was found to consist mainly of seeds, mainly from grasslands [14,15].

This study aimed to introduce novel ecosynthesis of AgNPs using pigeon dropping (PD) extract as eco-friendly agent. The physical and chemical features of synthesized AgNPs were confirmed by using different techniques and furthers tested as anti-bacterial, anti-fungal and anti-cancer agents.

## 2. Methodology and Materials

### 2.1. Preparation of Bio AgNPs

Raw dry pigeon droppings (PD) were obtained from a pigeon shed in a local farm hangar in Riyadh. Major organic particles such as large bones or whole eggs were avoided during collection. 17 g of PD has been converted to 90 mL of distilled water and is well blended by using a spoon for 3–5 min then soaked overnight for about 12 h. The extract was filtered through a gauze fist and then through a filter paper of Whatman No. 1 then stored at 4 °C until use. AgNP’s synthesis involved the addition of 0.012 g of AgNO_3_ to 50 mL of distilled H_2_O and was stirred and stored for 15 min at 90 °C. Next, 5 mL of PD extract was added. After 50 min, the color changed to light yellow and dark brown, indicating the formation of AgNPs. At 10,000 rpm, the reaction mixture was centrifuged for 15 min. After spinning down, the pellet was collected and washed three times by deionized H_2_O, then the resulting pellet was dried.

### 2.2. Characterization of Bio NPs

By using Shimadzu UV 2450 UV-Vis spectroscope (Shimadzu Corporation, Kyoto, Japan) over 230–850 nm, the synthesized NPs have been identified and characterized. The size of these NPs was detected by DLS technique through a zetasizer (Nano series, HT Laser, ZEN3600 Malvern Instruments, Malvern, UK). Characterization of NPs size, shape and morphology was performed by TEM (JEM-2100F, JEOL Ltd., Tokyo, Japan) at 200 kV accelerating voltage. Analysis of EDX was carried out using JEM-2100F TEM to detect AgNPs and other elementary components of the particles in suspension.

### 2.3. Assessment of Microbial Activity

The synthesized AgNPs antimicrobial activity was evaluated using the agar well-diffusion method [16]. In this study, four bacterial strains (*Escherichia coli*, *Pseudomonas aeruginosa*, *Staphylococcus aureus* and *Bacillus Sp*) and four fungal strains (*Aspergillus flavus*, *Penicillium griseofulvum*, *Alternaria alternate* and *Fusarium oxysporum*) were used. The bacteria examined are grown in blood agar (at 37 °C for 18 h) then the bacterial colonies were suspended 0.85% NaCl, adjusted to 0.5 MacFarland (108 CFU/mL) turbidity. The bacterial and fungal suspensions were smeared on Muller Hinton agar (MHA) plates and potato dextrose agar (PDA) medium, respectively. 100 µL of extract was loaded in the wells then incubation of bacterial and fungal plates at 37 ° C for 18–24 h and 28 °C for 48–72 h, respectively. After incubation, the plates exhibited clear zones of inhibition around the wells, which confirm the antimicrobial activity of bio AgNPs. The inhibition zone around each well was calculated by measuring its diameter.

### 2.4. Evaluation of Cytotoxic Effects

VACSERA Tissue Culture Unit has obtained the human breast (MCF-7) and lung cancer (A-549) cell lines. Dimethyl sulfoxide (DMSO) and trypan blue were purchased from Sigma, St. Louis, MO, USA. From Lonza, fetal bovine serum (FBS), Dulbecco’s modified Eagle medium (DMEM), HEPES buffer solution, L-glutamine, gentamycin, and 0.25% Trypsin-EDTA, were purchased. Using 10% heat-inactivated FBS, 1% L-glutamine, HEPES buffer and 50 µg/mL gentamycin, MCF-7 and A-549 was extended in DMEM. At 37 °C using 5% CO_2_ these cell lines are incubated and subcultured twice a week.

For the cytotoxicity assay, the cells they have been seeded at 1 × 10^4^ per well in 96-well plates. Serial two-fold dilutions of the tested AgNPs were added. The plates were then incubated for 24 h, a colorimetric method was used to determine viable cell yield. All experiments were carried out three times. The cytotoxic effect of each compound being studied and the standard reference drug was determined. A microplate reader (SunRise, TECAN, Inc, San Bruno, CA, USA) was used to calculate optical density (OD) to assess the percentage of viability. The 50% inhibitory concentration (IC_50_) was assessed using the GraphPad Prism program (San Diego, CA, USA) from the graphic plots of the dose response curve of each concentration [17,18].

## 3. Results and Discussion

### 3.1. Visual Examination and UV-Vis Spectral Analysis of Bio AgNPs

**Figure 1:**
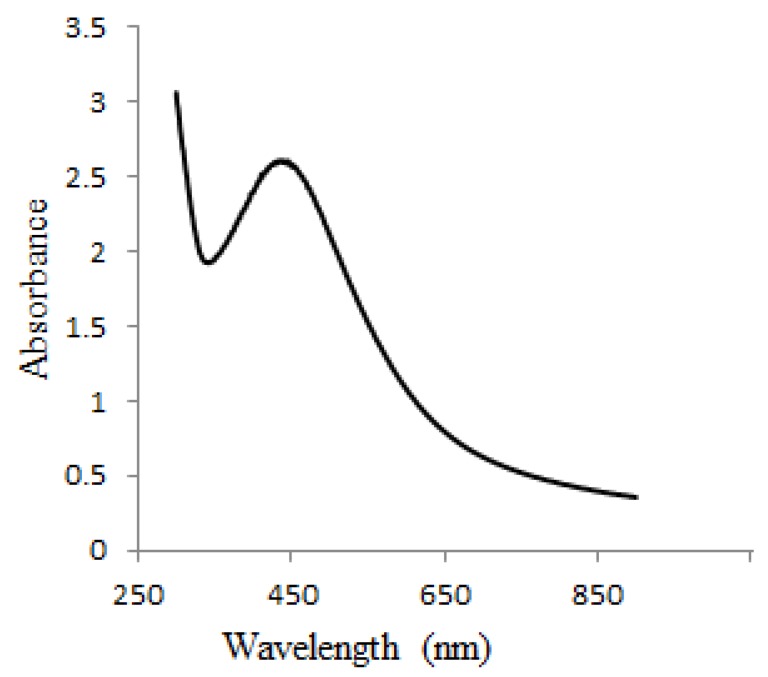
Absorption spectra of the synthesized bio silver NPs (AgNPs).

Metal NPs’ absorption spectrum is sensitive to various factors, including particle size, particle form and nano-particle interactions with the medium [19]. Using a UV-Vis spectrophotometer, it is possible to monitor the aqueous bio reduction of Ag^+^ ions effectively. The nucleation started very quickly and AgNPs were produced very fast until approximately 50–60 min. This was evident in the gradual increase in surface plasmon resonance (SPR) band intensity. The process then slowed down, and no further change in the SPR band was observed after 80 min. This result confirmed the completion of the reaction. AgNPs have free electrons, resulting in the SPR absorption band observed at 454 nm (Figure 1) due to the combined motion of metal NP electrons in resonance with light waves [20]. The broadening peak indicated poly-dispersed particles.

### 3.2. Particle Size Determination by DLS

**Figure 2:**
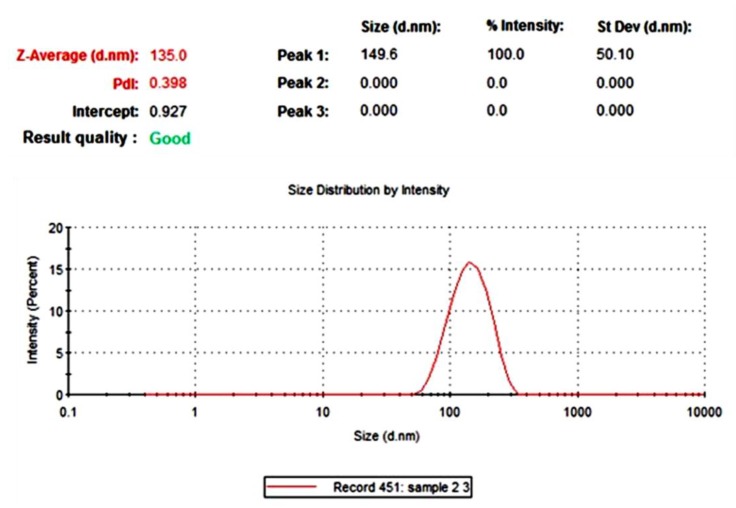
Zetasizer measurements of the average diameter (nm) of pigeon dropping (PD) AgNPs.

Particle size is used for characterizing NPs. PD AgNPs average particle size was 135 nm with 0.398 polydispersity index (PDI) and 0.927 intercept (Figure 2), which were observed clearly from the peak. This finding indicates that the particles are quite stable. This was supported by the fact that the time period between synthesis and bioassays was one month and the particles retained their efficacy. The most likely reasons for this change in hydrodynamic size include a small increase in the number of aggregates present and presence of a wide-ranging PD extract coating around the particles [21].

### 3.3. TEM Analysis of PD AgNPs

**Figure 3:**
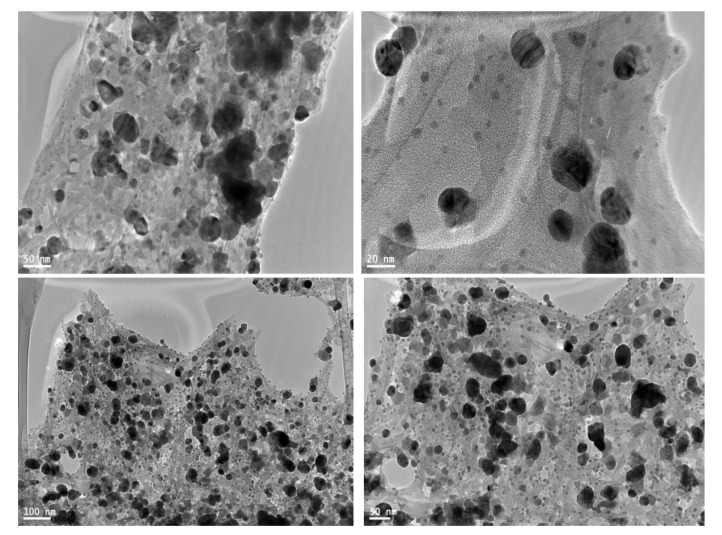
TEM images of the PD AgNPs.

TEM examined the morphology and size of AgNPs. AgNPs showed prominently spherical shape (Figure 3). The high-resolution TEM images also showed that AgNPs were not in physical contact but were separated by uniform distances. AgNPs capping was observed through a TEM micrograph, which may be due to the presence of bio-organic compounds in the PD extract. The small-sized NPs easily penetrated the membrane [22,23].

These findings are in consistent with the UV-vis spectrophotometric measurements, which show a broad SPR band due to the adsorption and aggregation of compounds in PD extract on the AgNPs surface (Figure 1). Other studies reported that AgNPs are coated with a small thin layer of other materials known to be capping organic materials in addition to fewer agglomerated particles [24,25].

### 3.4. EDX Analysis of PD AgNPs

**Figure 4:**
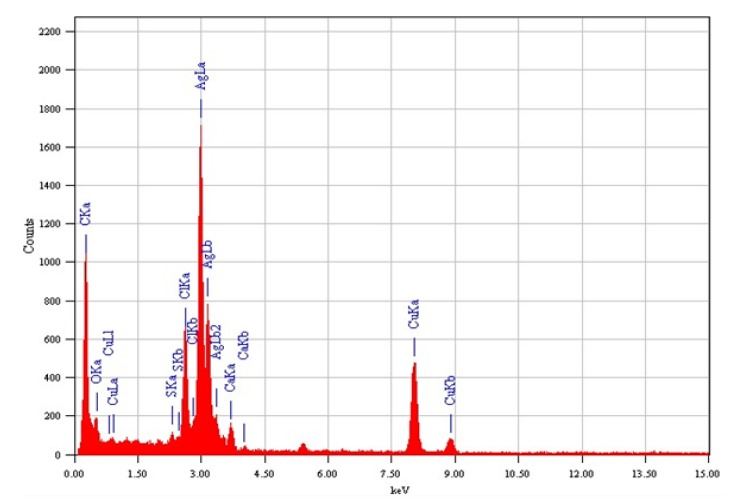
EDX spectra of PD AgNP suspension.

The analysis of the EDX showed that Ag is present in the suspension and it emits a strong signal, which confirms AgNPs formation (Figure 4). At approximately 3 KeV, AgNPs had a typical optical absorption peak due to SPR [26]. The EDX data also show a strong signal from Cu, which results from Cu TEM grid. Other elementary signals have been documented, possibly due to the presence of other elements within the PD extract.

### 3.5. Evaluation of Antimicrobial Activity of PD AgNPs

**Figure 5:**
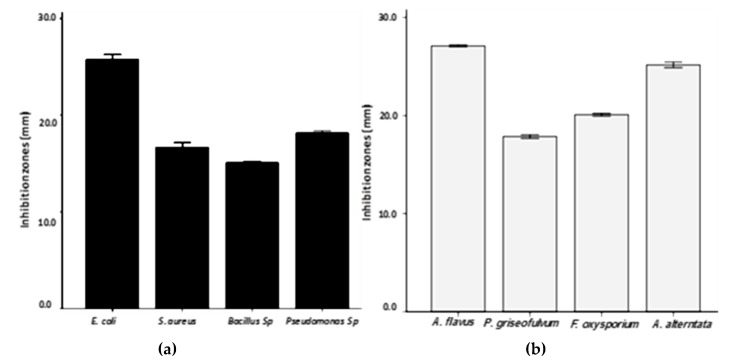
Demonstration of the antimicrobial activity of AgNPs synthesized from PD extract using agar well diffusion against pathogenic (**a**) bacteria; (**b**) fungi.

**Figure 6:**
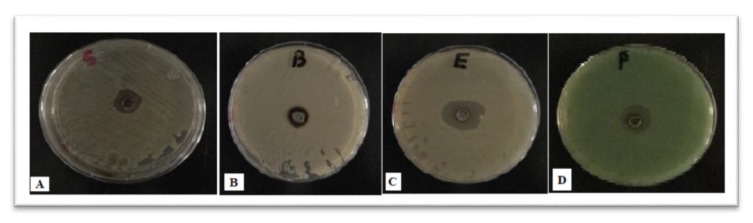
Plates showing the synthesized AgNPs antibacterial activity against (**A**) *S. aureus*, (**B**) *Bacillus,* (**C**) *E. coli, and* (**D**) *P. aeruginosa*.

**Figure 7:**
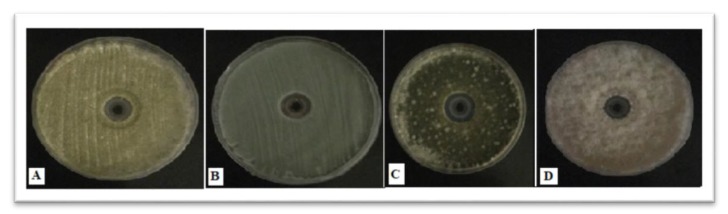
Plates showing the synthesized AgNPs antifungal activity against (**A**) *A. flavus*, (**B**) *P. griseofulvum*, (**C**) *A. alternate*, and (**D**) *F. oxysporum*.

The PD AgNPs showed their effects on both gram-positive *S. aureus* and *Bacillus* and gram-negative *P. aeruginosa* and *E. coli*. The antibacterial effects of the NPs are shown in Figure 5 and Figure 6. In addition, the resulting NPs showed that the inhibition zone was higher in gram negative than gram positive bacteria. The radial diameters of the inhibition zone for the obtained PD AgNPs were calculated to be 26 mm, 18 mm, 17 mm, and 15 mm for *E. coli*, *Pseudomonas aeruginosa*, *S. aureus*, and *Bacillus*, respectively. This is the first study according to the best of our knowledge that synthesized AgNPs using PD extract and evaluated their antimicrobial effects. Chikkanna et al., 2018 [27], studied the antimicrobial effects of AgNPs synthesized from fecal extracts from sheep and goat, and their results showed the antibacterial activity of the synthesized AgNPs for both strains of bacteria. Our results showed prominent antibacterial activity of PD AgNPs against all the organisms, and when compared to the findings of Chikkanna et al., the activity was less significant for NPs synthesized from goat and sheep fecal matter. This is possibly due to the capping of NPs with some organic compounds or some minute amounts of goat and sheep fecal matter leading to reduced activity of the NPs. Moreover, the PD consists mostly of the components of the seeds eaten by the pigeons, which were primarily obtained from grasslands. Furthermore, results revealed that PD AgNPs have highest antifungal effect against *A. flavus* and the lowest effect against *P. griseofulvum* (Figure 5 and Figure 7).

Previous studies have attempted to explain the mechanisms of AgNPs action as a bactericidal agent [27,28]. One of these studies suggested that due to their high surface area, AgNPs have effective antibacterial activity compared to the extract. This high surface area gives microorganisms a better contact [29]. The NPs bind to the cell membrane and enter the bacteria. The AgNPs interact with the proteins in the bacterial membrane containing sulfur and with compounds containing phosphorus such as DNA [30]. Other study found that when AgNPs enter the bacterial cell, they form an area of low molecular weight in the center of the bacteria, which conglomerates, protecting the DNA from the silver ions [31]. Another study suggested that the NPs inhibit cell division, which eventually leads to cell death, by preferentially attacking the respiratory electron transport chain [32]. Furthermore, the AgNPs might also exert their bactericidal effect via interaction with thiol group-containing respiratory enzymes of bacterial cells to inhibit the respiratory process [32]. In addition, it has been shown that AgNPs can enter the bacterial cells and condense the DNA, thereby preventing DNA replication and cellular reproduction [28].

### 3.6. Cytotoxicity Assessments of PD AgNPs

**Figure 8:**
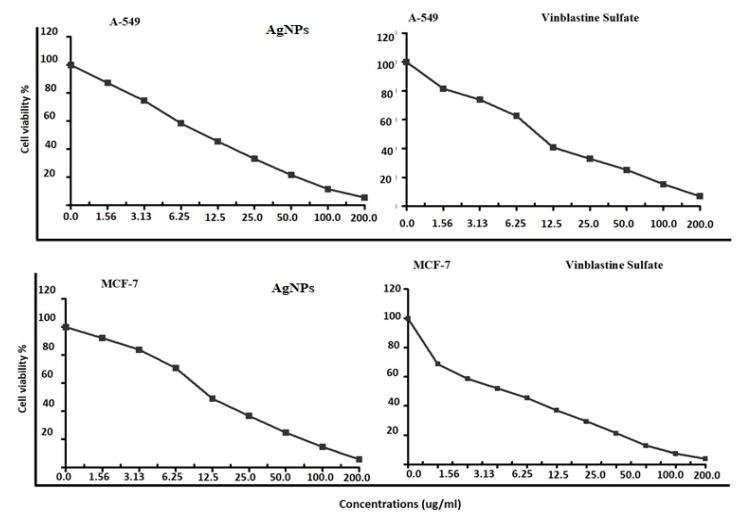
Cytotoxicity evaluation of PD AgNPs against A-549 and MCF-7 cell lines.

The cytotoxic properties of *PD* AgNPs against a lung cancer line (A-549) and breast cancer cell line (MCF-7) were determined in vitro. From the MTT assay, cytotoxicity against both cell lines increase with the increase of PD AgNPs concentrations. The NPs inhibited the proliferation of cells in a dose and time dependent manner. The IC_50_ of AgNPs and Vinblastine sulfate were detected at 10.3 ± 1.15 and 24.6 ± 0.7 µg mL^−1^ against A-549, respectively. The IC_50_ of AgNPs and Vinblastine sulfate were also detected at 12.19 ± 0.75 µg mL^−1^ and 5.11 ± 0.75 µg mL^−1^ against MCF-7, respectively (Figure 8). Regarding the cytotoxicity rates, Venugopal et al. obtained similar results [33], who found the IC_50_ of the AgNPs to be 60 µg mL^−1^ and 50 µg mL^−1^ for MCF-7 and A-549 cells, respectively. In this study, PD AgNPs cytotoxic effect against A-549 and MCF-7 cancer cell lines was promising and showed a potential in vitro anticancer activity. Recent studies with biosynthesized AgNPs have promising results with new and innovative therapies for different types of cancer, but some obstacles need to be addressed if these materials are to become clinically useful. A key point in anticancer treatment is the use of highly selective drugs or molecules in cancer cells to leave the surrounding tissues unaffected [34].

## 4. Conclusions

The study investigated AgNPs synthesized using waste material PD extract as a reducing agent. In the detailed characterization using UV-Vis spectroscopy, the NPs showed a characteristic plasmon absorption peak at 454 nm. The TEM images showed the sphericality of the NPs, and the elemental EDX analysis confirmed the formation of silver. These novel NPs exhibited potential antimicrobial and anticancer activities.
